# Diffuse idiopathic pulmonary neuroendocrine cell hyperplasia in a male patient associated with pulmonary adenocarcinoma

**DOI:** 10.36416/1806-3756/e20240211

**Published:** 2024-12-17

**Authors:** Murilo de Sá Barrêto Callou Peixoto, Augusto Kreling Medeiros, Felipe Marques da Costa

**Affiliations:** 1. BP Medicina Diagnóstica, Hospital Beneficência Portuguesa de São Paulo, São Paulo (SP) Brasil.; 2. Serviço de Pneumologia, Hospital Beneficência Portuguesa de São Paulo, São Paulo (SP) Brasil.

**Figure 1:**
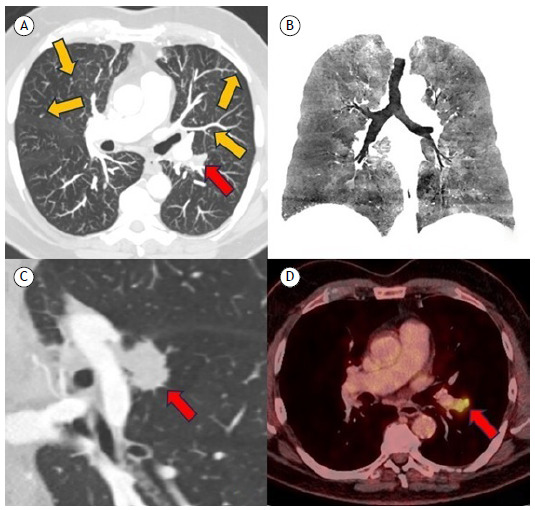
A: an axial CT image in lung window setting reformatted in maximum intensity projection depicts multiple lung nodules (yellow arrows) in line with the diagnosis of diffuse idiopathic pulmonary neuroendocrine cell hyperplasia (DIPNECH); the main lesion is shown in A (red arrow). In B, a coronal CT image in lung window setting reformatted in minimal intensity projection demonstrates mosaic attenuation in the lungs, commonly found in DIPNECH. In C, a coronal CT image in lung window setting shows the morphological characteristic of irregular margins of the main lesion (red arrow), which distinguishes it from the other lesions. In D, a fusion PET-CT image demonstrates focal glycolytic hypermetabolism in the lesion (red arrow); none of the other smaller nodules showed significant ^18^F-fluorodeoxyglucose uptake.

A 77-year-old hypertensive, diabetic, former-smoker male with a history of aortoiliac aneurysm repair presented with significant claudication and persistent productive cough, prompting evaluation. CT scans revealed multiple small pulmonary nodules (Figure 1A) and mosaic attenuation (Figure 1B), indicative of diffuse idiopathic pulmonary neuroendocrine cell hyperplasia (DIPNECH), and a concerning 3.0-cm mass (Figure 1C) in the superior segment of the lower left pulmonary lobe, with slightly irregular margins, suggestive of carcinoma considering the patient’s high-risk profile. ^18^F-fluorodeoxyglucose PET-CT showed exclusive radiotracer uptake in the suspected lesion, with a maximum standardized uptake value of 6.0 (Figure 1D). Lobectomy was performed, and the mass was confirmed as a mucinous adenocarcinoma (programmed death-ligand 1 = 0%), with no mutational drivers, accompanied by multiple foci of neuroendocrine hyperplasia. The radiologist’s expertise was crucial for distinguishing the imaging patterns of these lesions, highlighting the importance of radiological assessment in guiding clinical decisions.

Correctly differentiating DIPNECH nodules from malignancies is crucial to prevent misdiagnosis,^1^ ensuring appropriate staging and management. This particular case stands out given the established female predominance in the epidemiology of DIPNECH within existing literature.^2^ This underscores the necessity for radiologists to be vigilant for DIPNECH when evaluating male patients, despite the atypical gender presentation, when imaging phenotypes favor this condition, as in this case.
